# Economic Valuation of Health Care Services in Public Health Systems: A Study about Willingness to Pay (WTP) for Nursing Consultations

**DOI:** 10.1371/journal.pone.0062840

**Published:** 2013-04-23

**Authors:** Jesús Martín-Fernández, Mª Isabel del Cura-González, Gemma Rodríguez-Martínez, Gloria Ariza-Cardiel, Javier Zamora, Tomás Gómez-Gascón, Elena Polentinos-Castro, Francisco Javier Pérez-Rivas, Julia Domínguez-Bidagor, Milagros Beamud-Lagos, Mª Eugenia Tello-Bernabé, Juan Francisco Conde-López, Óscar Aguado-Arroyo, Mª Teresa Sanz- Bayona, Ana Isabel Gil-Lacruz

**Affiliations:** 1 Unidad Docente Multiprofesional de Atención Familiar y Comunitaria Oeste. Gerencia de Atención Primaria, Madrid, Spain; 2 Unidad de Apoyo a la Investigación, Gerencia de Atención Primaria, Madrid, Spain, Facultad de Ciencias de la Salud, Universidad Rey Juan Carlos, Madrid, Spain; 3 Centro de Salud Condes de Barcelona, Gerencia de Atención Primaria, Madrid, Spain; 4 Unidad de Bioestadística Cínica, Hospital Ramón y Cajal (IRYCIS) y CIBER de Epidemiología y Salud Pública (CIBERESP), Madrid, Spain; 5 Centro de Salud Guayaba, Gerencia de Atención Primaria, Madrid, Spain, Facultad de Medicina, Universidad Complutense de Madrid, Spain; 6 Unidad Docente Multiprofesional de Atención Familiar y Comunitaria Norte, Gerencia de Atención Primaria, Madrid, Spain; 7 Dirección Técnica de Procesos y Calidad, Gerencia de Atención Primaria, Madrid, Spain Facultad de Enfermería, Fisioterapia y Podología, Universidad Complutense de Madrid, Madrid, Spain; 8 Subdirección de Promoción de la Salud y Prevención, Dirección General Atención Primaria, Madrid, Spain; 9 Centro de Salud El Greco, Gerencia de Atención Primaria, Madrid, Spain; 10 Centro de Salud El Naranjo, Gerencia de Atención Primaria, Madrid, Spain; 11 Centro de Salud Laín Entralgo, Gerencia de Atención Primaria, Madrid, Spain; 12 Centro de Salud Francia, Gerencia de Atención Primaria, Madrid, Spain; 13 Dirección Asistencial Oeste, Gerencia de Atención Primaria, Madrid, Spain; 14 Escuela de Ingenieria y Arquitectura, Universidad de Zaragoza, Zaragoza, Spain; UNAIDS, Switzerland

## Abstract

**Background:**

Identifying the economic value assigned by users to a particular health service is of principal interest in planning the service. The aim of this study was to evaluate the perception of economic value of nursing consultation in primary care (PC) by its users.

**Methods and Results:**

Economic study using contingent valuation methodology. A total of 662 users of nursing consultation from 23 health centers were included. Data on demographic and socioeconomic characteristics, health needs, pattern of usage, and satisfaction with provided service were compiled. The validity of the response was evaluated by an explanatory mixed-effects multilevel model in order to assess the factors associated with the response according to the welfare theory. Response reliability was also evaluated. Subjects included in the study indicated an average Willingness to Pay (WTP) of €14.4 (CI 95%: €13.2–15.5; median €10) and an average Willingness to Accept [Compensation] (WTA) of €20.9 (CI 95%: €19.6–22.2; median €20). Average area income, personal income, consultation duration, home visit, and education level correlated with greater WTP. Women and older subjects showed lower WTP. Fixed parameters explained 8.41% of the residual variability, and response clustering in different health centers explained 4–6% of the total variability. The influence of income on WTP was different in each center. The responses for WTP and WTA in a subgroup of subjects were consistent when reassessed after 2 weeks (intraclass correlation coefficients 0.952 and 0.893, respectively).

**Conclusions:**

The economic value of nursing services provided within PC in a public health system is clearly perceived by its user. The perception of this value is influenced by socioeconomic and demographic characteristics of the subjects and their environment, and by the unique characteristics of the evaluated service. The method of contingent valuation is useful for making explicit this perception of value of health services.

## Introduction

Definition of health policies must be accompanied by the knowledge of the preferences of the citizens who will benefit from them, since this has been shown to be related to efficiency and quality of health care [1]. A key element in defining health policies is the organization of the system of health service provision and the distribution of resources among these different provided services. In our country, Spain, health care is organized as a national health system. The service provision is organized into two levels, Primary Care (PC) and Specialized Care (SP). PC has been defined as the gatekeeper of the health system and, even though it has managed the health needs of patients for 30 years with a high degree of satisfaction [2], it is suffering from progressive disinvestment during the last years in comparison with the rest of the health organization [3], [4]. This trend to reduce investment in PC is not justified by an analysis of social need as expressed by health demand since demand for PC is high, and even higher among those with a poorer perception of their health condition [5]. This situation cannot be justified by means of economic rationale either, since it is known that investment in PC leads to more efficient health outcomes [6]. Knowledge on patients' preferences with respect to distribution of health resources, together with the rest of mentioned factors, namely health needs and economic rationale, can contribute to improving the process of planning health systems.

The opinions expressed by users of health systems about their preferences can be studied from several perspectives. One way of approaching the problem, which provides comparable results with expressed preferences for services or goods of different nature, is to study the perception of economic value. Specific methodologies exist to study the economic value of non-market goods, such as Contingent Valuation (CV). The CV method, which is based on economic theory and was developed into cost-benefit analysis (CBA), attempts to simulate a hypothetical market by surveying consumers with questionnaires. The objective of the questionnaire is to build a hypothetical scenario where interviewed subjects represent the demand and the interviewer plays the role of supply. In comparison with other evaluation techniques, such as cost-effectiveness analysis, and similarly to cost-benefit analysis, the CV method, has the advantage that benefits for a program or service can be compared directly with costs because they are both in monetary units. The CV method assumes that preferences of individuals can be interpreted in the form of a utility function, where two states (initial and final) can be compared in terms of the changes of the utility function. If people apply their preferences under a social point of view, we could estimate the social welfare function defined by the sum of individual perceptions. The CV method is particularly appropriate when valuing health care because of its ability to include both use and non-use values, that is, the value derived from considering the service as a commodity and the value as a public good [7]. This methodology, whose strengths and limitations have been widely discussed [8], has been utilized in the context of community health care. It has been used to estimate WTP to improve health care in developing systems [9], and for valuation of programs of health promotion [10], mental health care [11], improvements in health condition derived from physical activity programs [12], or WTP for formal [13] and informal [14] care.

The perception of a value of a service can be studied for the whole service or for the elements that comprise it. The basic care pillar in PC is the Basic Care Unit, also called Family Care Unit, composed of a family physician and a family or community nurse. It is known that, in our setting, users of family medical care consultation perceive an economic value for the service, despite the lack of a direct payment at the moment of using it, called “price signal” [15]. However, there is no available information regarding the users' perception of value of nursing services in PC. Knowing the monetary value that users of a health system assign to a specific service provides useful information for deciding the investment level and the “quantity of need” that should be addressed, since it establishes an unequivocal expression of the users' own preferences.

In this work, we intended to evaluate the perception of economic value expressed by users of nursing services in PC, the validity of the measured parameters by assessing their agreement with the economic theory of welfare, and their reliability. The validity of the results was also studied within the context of this theoretical framework [16], according to which it is expected that subjects with a higher income, those more satisfied with the product, or those who made more use of it, would perceive a greater value for the evaluated service.

## Methods

### Design

This study employed economic valuation, using the Contingent Valuation (CV) method to estimate Willingness to Pay (WTP) and Willingness to Accept [Compensation] (WTA) for the health care service received during primary care (PC) nursing consultations, within the public health system of the Community of Madrid.

### Subjects

The criteria of inclusion were: 18 years of age or older, properly understanding Spanish, previous experiences in goods exchange within market conditions in order to be able to understand the posed scenarios, and written consent to be interviewed. The criteria of exclusion were: not understanding the language, not being qualified to give consent, or not understanding the purpose of the questionnaire.

The centers were selected in the Community of Madrid. This is one of the seventeen autonomous communities (regions) of Spain. It is located in the middle of the country and it is a community with only one province. Its capital is the city of Madrid, which is also the national capital of Spain. It has a population of 6,369,167 (2011), more than 80% concentrated in the city of Madrid and its metropolitan area. The selection of the health centers was carried out so that both rural and urban environments were represented, as well as the upper and lower terciles of the income distribution in the Community of Madrid. Twenty-three centers were included, from 6 of the 7 areas in which PC of the CM is administratively divided. Of these, 6 centers were located in rural areas and 17 in urban areas (inside the city of Madrid, the capital, or its metropolitan area), 12 belonged to high-income areas and 11 to low-income areas. Within each center, systematic sampling of attendance records was used to select the subjects, obtaining 90% of patients from in-center consultations and 10% from home visits.

### Sample size and recruitment

Sample size was calculated in order to estimate WTP and WTA parameters, so that the range of the confidence interval should not be more than 30% of the standard deviation (precision 15%), for a confidence limit of 95%. Using the standard formula for this calculation [17], 170 subjects needed to be included. Since groups of subjects were going to be studied (patients from the same health centers), where the variability of the parameter to be studied would be smaller among the subjects from the group and greater among subjects from different groups, the design effect was estimated in order to be able to correct for this in our calculation. In a previous study to evaluate WTP per medical consultation, the value of the intraclass correlation coefficient (ICC) was 0.048 [15]. We estimated a more adverse scenario where ICC is 50% higher (ICC≈0.075) which, when including approximately 30 patients per center, yields a design effect of 3.18 [18], and requires inclusion of 543 subjects (170×3.18). Since there would be a percentage of incomplete questionnaires, or patients not revealing the necessary data for later analysis, and given a small marginal cost per extra questionnaires, our objective of inclusion was 600 patients. This meant recruiting around 30 patients from 20 health centers.

### Measurement tools and included variables

The CV method requires individual questionnaires to estimate WTP and WTA. In all cases, the interview was performed at the time patients exited the nursing consultation by the same interviewer, previously trained and knowledgeable in the method, at a location within the health center but outside the care area. In the case of home visits, the interview was done over the telephone, a format previously tested and used in other studies [11], [19].

The monetary measure of the change in the communicated utility will refer to a compensatory variation framework of the classification proposed by O'Brien and Gafni (compensation required so that, following the change, the utility remains the same as before the change) [16].

The format used for the payment was direct payment through a “payment card”, as it is assumed that this allows the user to behave as they would in a context where a product is sold at different prices [20]. After presenting the scenario, the following question was used to determine WTP: *“Imagine you have a similar health need to the one that brought you to the consultation today, and you must be attended by the same nurse who attended you today, but you have to pay for that service directly; how much would you be willing to pay for this consultation?”* In order to calculate WTA, the following question was used: *“[…] it was decided not to provide the service in the manner it has been provided until now [public health service, free access] and to compensate the citizen who will receive a check for the loss of the service. What would be the minimum quantity that you would require to receive in order not to feel harmed by the loss of this specific service?”*

Answers were collected through a system of payment cards in 2 phases. The first card contained 3 possible values: less than €20, €20–40, and more than €40. The second payment card contained the following values: €0, €5, €10, €15, €20, €25, €30, €35, €40, €45, €50, €55, €60 and more than €60. The answer to the second payment card had to be consistent with the first answer. If an answer of €0 was stated, the patient was asked for the reason, using a multiple-answer question with 4 possible answers: “I cannot afford to pay for this service”, “I am not willing to pay for this service”, “I do not find this question to be pertinent”, and “Other reasons”. If the answer was “over €60”, the subject was asked to provide the exact value. The values of the first payment card were chosen after a pilot study of the questionnaire in a group of 19 health professionals and patients.

To be able to validate the answer given for the perceived value, variables grouped by characteristics of the health center, of the consultation, and of the patient were collected.

The health center characteristics depended on its urban or rural environment and on the available data regarding the area's average income, classified as upper and lower tercile (based on data from 2008, Statistics Institute, Community of Madrid). The characteristics of the consultation were: the place of assistance (in-center or home visit), type of service (preventive, chronic disease follow-up, care plans follow-up, or diagnostic and therapeutic procedures), time elapsed to obtain the appointment (same day, or 1, 2, 3, or >3 days), waiting time at the center prior to being attended (<15, 16–30, 31–60, or >60 minutes), perceived duration time of consultation (<1, 1–5, 6–10, 11–15, 16–30, or >30 minutes), and origin of consultation (patient initiated, self-appointment by the nurse, initiated by different nurse, initiated by other health professionals). Demographic and socioeconomic characteristics of the included patients were: age, sex, nationality, education level (illiterate, no education, primary education, secondary education, superior education), “social class” in a 6-category classification [21], family income (in thousands of euros adjusted by the number of people per household, as proposed by the Organisation for Economic Co-operation and Development), and patient perception of family support measured by the Apgar test [22].

With respect to health needs and utilization of health services, we studied the existence of chronic pathologies (those requiring continued health care for >6 months), hospital admissions during the previous year (including emergency room stays >24 hours), number of medical and nursing consultations during last year, and perception of health condition measured by EuroQol-5D. The results of EuroQol-5D were expressed in the visual scale and transformed into analog as proposed by Herdman et al. [23]. Subjects were also asked about the existence of other insurances as an indirect payment for health services (and not only through taxes).

To evaluate satisfaction with the provided service, a questionnaire validated in our setting was used to measure 3 dimensions: care provided by the health professional, consultation duration, and depth of the relationship with the health professional [24].

Since WTP is subject to the patient's ability to pay, as has been shown in previous studies [10], [13], [15], [25], adjusted family income was the main independent variable chosen.

### Data analysis

The descriptive analysis was expressed by measures of central tendency and dispersion and by their 95% confidence intervals. Median and interquartile ranges were used in cases of asymmetric distributions.

Several explanatory models were built in which the dependent variable was the WTP, specifically represented by the smoothed function ln (WTP) (natural logarithm of WTP) due to its asymmetric distribution. Multilevel models were chosen as they allow studying aggregated data, evaluating not only the explanatory capacity of the variables, but also the influence of groups in the variability of the measures [26]. Every model consisted of two levels, the individual and the group they belong to (the health center in this case), and was expressed using a generic function of the form:
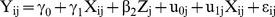
Where Yij is the natural logarithm of the expressed WTP, X_ij_ represents the variables of each individual “i” from the “j” group, Z_j_ is the whole of variables in each group “j”, u_0j_ is the random effect of the mean of each group, u_1j_ is the random effect of the slope of each group “j”, and ε_ij_ is the random error of an individual “i” from the “j” group.

The main independent variable chosen was the adjusted family income, as it can represent the ability to pay, while the rest of the variables were studied as covariables. This variable was used in the model in its “centered” form, that is to say, it was obtained by subtracting the mean from every value of the distribution 

Three models were built. Model 1, termed “empty model”, was built without explanatory variables, and represented the variability of the answer, WTP, in the studied health centers. The second model included all the explanatory variables as fixed effects and the random effects, in order to assess the association of different variables with WTP and whether the mean of WTP was different in each center. The explanatory variables were introduced in the model in an attempt to maximize its explanatory capability and in accordance with the parsimony principle. The third model studied the variability of the slope of the main variable among different groups, that is, if the relationship between the adjusted family income and WTP were different in different centers.

STATA software version 12 was used for statistical analysis and Restricted Maximum Likelihood (REML) methods were employed to build the models.

To evaluate the reliability of the answers, the questionnaire was repeated on the telephone to 1 of every 5 subjects included in the study and the ICC was calculated (absolute agreement) for the initial and final measures of WTP and WTA.

### Ethical and legal aspects

The entire research process has been governed by the ethical principles contained in the Declaration of Helsinki (Sixth Revision, Seoul 2008). Included patients were asked for written consent to participate in the study. All the information was processed and subsequently stored in an anonymous way, accomplishing the requirements established in national legislation. The study relies on the favorable report by the Ethics Review Board of the Hospital Universitario Fundación Alcorcón, Madrid, Spain.

## Results

Seven hundred and fifty-seven subjects were asked to take part in this study, out of which 95 declined to participate (12.6% of total, CI 95%: 10.1–15.0%). The reasons for not participating were: lack of time (63), lack of interest in the study (12), other reasons (11), and no provided reason (9). The 95 subjects who declined to participate differed from the 662 subjects included in study only in the type of obtained consultation, which more frequently consisted of diagnostic and therapeutic procedures (78.7% of cases, CI 95%: 69.9–87.5%). Table 1 shows the characteristics of the studied population and table 2 shows the characteristics of the service received by the patients included.

**Table 1:** Characteristics of subjects included in the study.

	Mean (CI 95%)	Median (IQ range)	Percentages (CI 95%)	
**Age (years)**	65.4 (64.1−66.6)	69 (55−78)		
**Sex (female)**			60.7% (56.9−64.5%)	
**Spanish Nationality**			95.2% (93.5−96.9%)	
**Other insurance**			16.3% (13.4−19.2%)	
**Chronic condition**			82.9% (79.9−85.9%)	
**VAS-EuroQol-5D**	65.6 (63.9−67.4)	70 (50−80)		
**EuroQol-5D Utilities**	0.68 (0.66−0.71)	0.76 (0.48−1.00)		
**Nursing consultations/year**	16.6 (14.6−18.7)	10 (5−16)		
**Hospital admissions in the last year**			29.2% (25.7−32.8%)	
**Family APGAR**	8.7 (8.6−8.9)	10 (8−10)		
**Education level:**				
** Illiterate**			3.9% (2.4−5.5%)	
** No education**			24.5% (21.1−27.8%)	
** Primary education**			34.1% (30.5−37.8%)	
** Secondary education**			23.4% (20.1−26.7%)	
** Superior education**			14.0% (11.3−16.7%)	
**Social group**				
** Manager, director**			9.1% (6.8−11.3%)	
** Intermediate positions**			13.3% (10.6−16.0%)	
** Skilled non-manual worker**			26.3% (22.9−29.7%)	
** Skilled manual worker**			23.0% (19.7−26.2%)	
** Partially skilled manual worker**			11.3% (8.8−13.8%)	
** Unskilled manual worker**			17.1% (14.1−20.0%)	
**Adjusted family income** * **(€1,000)**	0.873 (0.833−0.912)	0.707 (0.600−1.000)		

CI 95%: Confidence interval 95%.

IQ Range: Interquartile range (25–75 percentile).

VAS-EuroQol-5D. Visual Analog Scale of EuroQol-5D questionnaire.

Family APGAR : Scores under seven point suggest dysfunctional family.

* Adjusted by means of the Organisation for Economic Co-operation and Development (OECD), proposed methodology.

**Table 2:** Characteristics of the consultation and subject's perceptions.

	Percentages (CI95%)	Mean (CI 95%)	Median (IQ range)
**Type of consultation**			
** Preventive activities**	12.5% (9.9−15.1%)		
** Chronic disease follow-up**	21.3% (18.1−24.5%)		
** Care plans follow-up**	2.6% (1.3−3.8%)		
** Diagnostic and therapeutic procedures**	63.6% (59.9−67.3%)		
**Who demanded the appointment**			
** Patient**	24.3% (21.0−27.7%)		
** Attending nurse**	65.4% (61.7−69.1%)		
** Derived from another nurse**	0.5% (0.1−1.3%)		
** Derived from other proffesionals**	9.8% (7.5−12.2%)		
**Time until appointment**			
** Same day**	81.3% (78.2−84.3%)		
** 1 day**	6.3% (4.4−8.3%)		
** 2 days**	4.8% (3.1−6.5%)		
** 3 days**	1.8% (0.7−2.9%)		
** >3 days**	5.7% (3.9−7.6%)		
**Waiting time at consultation**			
** <15 minutes**	84.9% (82.1−87.7%)		
** 16–30 minutes**	12.1% (9.5−14.6%)		
** 31–60 minutes**	2.65 (1.3−3.8%)		
** >60 minutes**	0.5% (0.1−1.3%)		
**Perception of consultation duration**			
** <1 minute**	0.6% (0.2−1.5%)		
** 1–5 minutes**	18.1% (15.2−21.1%)		
** 6–10 minutes**	42.4% (38.6−46.3%)		
** 11–15 minutes**	23.0% (19.7−26.2%)		
** 16–30 minutes**	12.5% (9.9−15.1%)		
** >30 minutes**	3.3 (1.9−4.8%)		
** Consultation duration**		13.3 (12.7−13.9)	10.0 (10.0−15.0)
**Satisfaction- overall** *		4.89 (4.85−4.92)	5.00 (5.00−5.00)
**Satisfaction- received care** *		4.76 (4.72−4.80)	5.00 (4.75−5.00)
**Satisfaction- dedicated time** *		4.48 (4.43−4.54)	5.00 (4.00−5.00)
**Satisfaction- relationship with health professional** *		3.34 (3.23−3.45)	3.67 (2.33−4.67)

CI 95%: Confidence interval 95%.

IQ Range: Interquantile range (percentile 25-percentile 75).

* 1 worst possible score, 5 best possible score.

Of the total of interviewed subjects, 81 people (12.2%) stated a WTP of €0. Out of these, 63 (77.8%) indicated inability to pay, 17 (21.0%) declared unwillingness to pay for the service, and 1 (1.2%) did not find the question to be pertinent. People expressing a WTP equal to €0 did not show any difference in the number of consultations or the perceived satisfaction, even though they had a lower adjusted family income (€−169.1, CI 95%: €−159.5 to −178.7). When asked about their WTA, 14 people (2.1%) did not provide any answer and 13 subjects (2.0%) gave an answer of €0. Of those who stated a WTP greater than €0, only 2 did not answer the question about WTA and other 2 gave it a €0 value. Table 3 shows the distribution, in percentile, of WTP and expressed WTA. WTP shows a mean of €14.4 (CI 95%: €13.2–15.5), and a median of €10. WTA, on the other hand, has a mean of €20.9 (CI 95%: €19.6–22.2) and a median of €20.

**Table 3:** Willingness to Pay (WTP) and Willingness to Accept [Compensation] (WTA) expressed.

	Percentile 10	Percentile 25	Percentile 50	Percentile 75	Percentile 90	Mean* (CI95%)	Mean** (CI95%)
**WTP (€)**	0	5	10	20	30	14.4 (13.2−15.5)	16.4 (16.4−17.7)
**WTA (€)**	5	10	20	30	40	20.9 (19.6−22.2)	21.3 (20.0−22.7)

* Including the 662 responses.

** Excluding €0 values (581 DAP responses and 635 DAC responses).

The explanatory model was built using ln (WTP) as the dependent variable, so only those subjects stating a WTP higher than €0 were included. All 3 models described in the data analysis section are presented.

A variability of 6.56% in the value of ln (WTP) can be attributed to clustering by health center (model 1, tables 4 and 5).

**Table 4:** Description of the explanative models: fixed effects.

Model	Parameter	Coefficient	P value	CI 95%
**Model 1** *	Constant	2.509	<0.001	2.412−2.606
**Model 2** **	Constant	2.328	<0.001	1.986−2.669
	High-income	0.198	0.019	0.033−0.363
	Home visit	0.227	0.036	0.015−0.439
	Duration of consultation (min)	0.011	0.020	0.002−0.020
	Relationship with health professional (1–5)	0.057	0.008	0.015−0.099
	Age (years)	−0.007	<0.001	−0.011−−0.003
	Sex (female)	−0.188	0.002	−0.305−−0.070
	Secondary or superior education	0.183	0.012	0.040−0.326
	Centered adjusted family income (€1,000)	0.127	0.038	0.007−0.246
**Model 3** ***	Constant	2.233	<0.001	1.991−2.674
	High-income	0.194	0.022	0.028−0.360
	Home visit	0.224	0.039	0.012−0.435
	Consultation duration (min)	0.011	0.020	0.002−0.020
	Relationship with health professional (1–5)	0.057	0.008	0.015−0.099
	Age (years)	−0.007	<0.001	−0.011−−0.003
	Sex (female)	−0.188	0.002	−0.305−−0.070
	Secondary or superior education	0.183	0.012	0.040−0.325
	Centered adjusted family income (€1,000)	0.122	0.069	−0.009−0.254

* Deviance −632.7. Significance for the Chi^2^ test <0.001.

** Deviance −618.5. Significance for the Chi^2^ test <0.001.

*** Deviance −618.3 Significance for the Chi^2^ test <0.001.

CI 95%: Confidence interval 95%.

**Table 5:** Description of the explanative models: random effects.

Model	Parameter		Estimation	CI 95%	Sig. (Chi^2^)	ICC
**Model 1**	Constant	Variance	0.036	0.014−0.091	<0.001	0.0656
	Residual	Variance	0.511	0.454−0.575		
**Model 2**	Constant	Variance	0.019	0.006−0.063	0.005	0.0396
	Residual	Variance	0.468	0.415−0.527		
**Model 3**	Income dependent	Variance	0.014	3×10^−4^−0.543	0.029	
	Constant	Variance	0.020	0.006−0.064		
	Residual	Variance	0.464	0.411−0.524		

CI 95%: Confidence interval 95%.

Sig (Chi^2^): Significance for the Chi^2^ test.

ICC. Intraclass Correlation Coefficient.

After introducing fixed effects into the model, the variability of ln (WTP) attributed to clustering is 3.96% relative to 6.56% in the empty model. This further explains a residual variability of 8.41% (model 2, tables 4 and 5). The following variables were excluded from this model for not improving its explanatory capacity: rural or urban environment, type of consultation, time elapsed to get an appointment, waiting time at the health center, solicitor of consultation, nationality, family support, existence of chronic pathologies, hospital admissions in the last year, number of consultations in the last year, and perception of health condition.

When looking at the fixed effects, and taking into account that the response variable is ln (WTP), it can be inferred that living in high income areas increases WTP by a mean of 22% (CI 95%: 3–44%). With respect to the characteristics of the consultation: WTP for home visit consultation has a 25% higher mean (CI 95%: 2–55%), every increase of 10 minutes in consultation duration produces an 11% higher WTP (CI 95%: 2–22%), and every increase of one point on the satisfaction scale in the relationship with the health professional increases WTP by a mean of 6% (CI 95%: 2–10%). Women express a mean WTP that is 17% lower than men (CI 95%: −7 to −26%), and for every additional 10 years of age the WTP decreases by 7% (CI 95%: −3 to −11%). Subjects with a higher level of education show a 20% higher mean WTP (CI 95%: 4–39%). Finally, for every €1,000 of higher adjusted family income versus the mean, average WTP increases by 14% (CI 95%: 1–28%).

In order to describe the combined influence of the explanatory variables, if we compared the expected WTP for a female, 70 years of age, lacking education, attending a 10 minute consultation with a satisfaction score of 1 in the proposed scale, with an adjusted family income of €600, and who lives in a low income area, with the expected WTP for a male, 40 years of age, with superior education, home visited for 30 minutes, with a satisfaction score of 5 in the same scale, with an adjusted family income of €2,600, and from a high income area, the average expected WTP would be 5.5 times higher in the second case.

Finally, model 3 (tables 4 and 5) is presented, where it is tested whether the slopes of the independent main variable (centered adjusted family income) differs among the groups.

**Figure 1:**
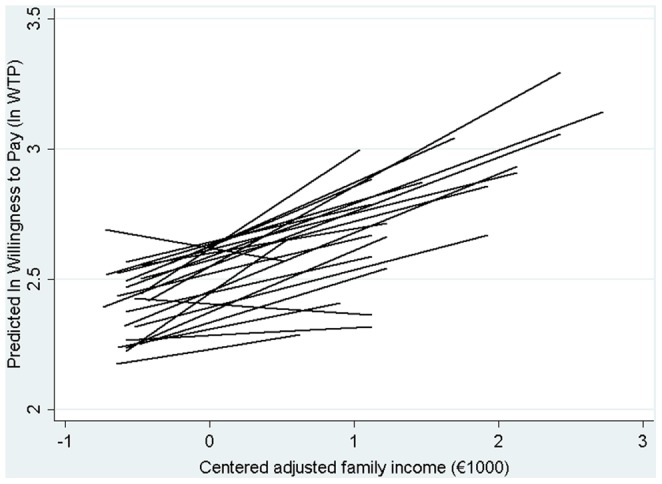
Expected adjusted association between adjusted family income and lnWTP in each center, variability of the slopes (model 3).

Previously, the existence of covariance between the mean WTP in each group and the slope of the income variable was hypothesized; however, this was ruled out (data not provided). The magnitude and significance of the response grouping are similar to those of model 2, confirming that the mean ln (WTP) is different in each group, and it is observed that the influence of family income changes in each group as well, since its slope varies from group to group (figure 1).

To calculate the part of the variability due to clustering in model 3, we must fix specific values of the independent variable since the slope is different in every group. This way, considering mean values of family income, the model's ICC is 4.10%, similar to that of model 2. As for values far apart from the mean (percentiles 25 and 75 of the family income distribution, €600 and €1,000 respectively), the effect of the clustering is more marked, having an ICC value of 4.29% and 4.12% for an income of €600 and €1,000 respectively. Only in extreme values of family income (€1,000 above the mean), the variability due to grouping, measured by the model's ICC, approaches similar or higher values than those of the empty model (6.85%).

**Figure 2:**
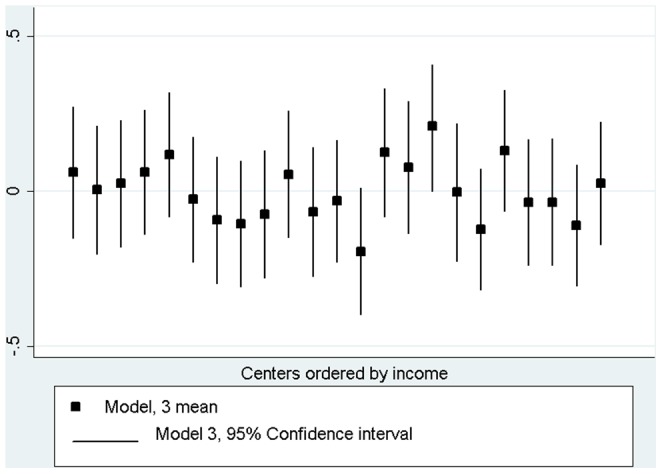
Bayesian residuals of each center in model 3 (ordered by mean income of the area).

Figure 2 shows bayesian residuals of model 3 that allow us to check the homogeneity of its distribution and the part of intergroup variability that remains unexplained.

In order to estimate ICCs for the expressed WTP and WTA, we used 127 pairs of measures for WTP and 125 for WTA. The ICC of the answer for WTP was 0.952 (CI 95%: 0.932–0.966) and 0.893 for WTA (CI 95%: 0.850–0.924).

## Discussion

The presented data allow us to assure that the studied population has a clear perception of the economic value for the received service at the nursing consultation, despite the lack of explicit payment at the moment of use. This perception seems to depend on personal characteristics of the subject, characteristics of the consultation, and, to a certain degree, the characteristics of the environment where the consultation takes place. Finally, it seems that the expression of perceived value using CV methodology is reliable.

As for the value of the WTP, the perception seems to be quite realistic, having a reported mean of €14.4 (median €10), which represents 80% of the value established by the Community of Madrid when re-invoicing other entities for nursing consultation (€18 in 2009, ORDER 629/2009, of August 31st B.O.C.M. Num. 215, September 10th 2009). Although the expressed value outcomes of WTA are usually consistently higher than those of WTP [27], [28], as is the case in this study (mean €20.9, median €20), use of WTP has been recommended as the main outcome of the contingent valuation since it appears to better reflect value perception and is more easily understood by the studied subjects [25]. If we compare the mean expressed WTP with the mean adjusted family income, we observe that WTP per consultation would represent 1.6% of that value, comparable to the 2% observed in our setting for the family physician consultation [15].

The characteristics explaining variations in the obtained answers are those expected a priori within the frame of the economic welfare theory. It was expected that subjects with higher payment ability and those who live in high-income neighbourhoods would show higher WTP. The role of financial capability as an explanatory factor of WTP was anticipated from the theoretical point of view [16], [25] and it repeatedly appears in the literature [9], [10], [11], [12], [13], [14], [15], [29], corroborating that the model is consistent with economic theory. The education level of subjects has been associated with higher WTP in studies of health services [15], [30]. Profiles of young, highly educated men have shown a higher WTP for both health services [9], [15] and for the outcome of health interventions [31].

Out of the studied variables, educational level was chosen as the explanatory variable for the model, and not “social class”, because the correlation of the latter with available income data was greater than that of the educational level and prejudiced global explanative capacity by increasing the co-linearity. Moreover, social class by itself strongly correlates with WTP for the studied service, as has been observed in other studies [13], [15].

The negative correlation between female gender and age with WTP for health services and products has been referred to in the literature [16], [31]. However, the role of gender in WTP for health services is contradictory [14], [30], with a higher WTP observed in certain occasions [32]. What does seem clear is that both women and elders show a higher economic elasticity for price increases in health services (they would consume a product less if its price increased) [9].

Visit duration and location are the consultation characteristics that are related to WTP. This is consistent with the theoretical framework that the greater the quantity of product received, and under more favorable conditions for the user, the higher their WTP will be. The same can be said about satisfaction. It is expected that the greater the satisfaction the higher the WTP, as has been observed using the scale measuring satisfaction in the relationship with the health professional.

Variables found to not be explanatory are those related to rural/urban environment of the center, nationality, accessibility, and type of consultation. However, regarding accessibility, it must be pointed out that more than 80% of interviewed subjects obtained the appointment the day of requesting it and had a waiting time prior to being attended of <15 minutes.

The lack of association between some variables correlating to health needs and WTP are difficult to interpret. In the chosen model, there does not appear to be any correlation between the number of chronic pathologies, hospitalizations, or the perception of their own health condition as measured by EuroQol-5D, by subjects and their expressed WTP. This fact may be partially explained by the way need is measured in the health setting, by the high percentage of patients with “chronic conditions”, or by the higher probability of selecting patients having more frequent consultations. On the other hand, both measures of health related quality of life by EuroQol-5D and chronic conditions are well correlated with age and adjusted family income (inversely correlated). This situation could cause a collinearity problem, which could lack signification for these variables in the explanatory model.

Nevertheless, some indirect indicators of health need, such as the necessity for consultations to be held at home (patients with mobility issues), have been clearly associated with WTP.

The reliability of WTP and WTA expressions had largely not been studied in health care literature and there was little information about test-retest reliability [25]. Once the validity of the response has been tested, the observed high test-retest reliability highlights the solidity of the method to elicit preferences, as far as the answers remain the same when asking for the same service in a short time period.

### Limitations

The limitations of this study are determined by the employed method. They have been correlated with the validity of the response and its reliability [8]. The validity of the response can be affected by the possibility of the presence of biases, derived from the construction of the study or its interpretation by the included subjects. The convenience of using the payment card as the form of answer has been a subject of discussion because, among other reasons, the possibility of inducing biases by providing an answer range [33]. In order to minimize possible biases that may be induced by the range of the payment card, the question was designed in 2 phases, the first providing a broad range and the second trying to narrow down WTP/WTA value.

Other biases such as the existence of a possible strategic bias, namely the one that leads to undervaluing a good when the subject believes the final price can get lowered, cannot be evaluated with certainty. This is becoming particularly relevant at the present time, when the co-payment of health services is a debated issue in the whole country. The percentage of zero value answers (12.2%) may be influenced by this fact, even though this percentage is similar or even inferior to that observed in literature when assessing WTP for health services [30], [32]. Existence of the hypothetical bias, which leads to overvaluing a good before having to pay for it in reality, must be considered as well. This can happen when the good is not well known (ex-ante studies), or when it is thought that the service will not be paid for in reality. The methodology used in our work (ex-post), the current social debate on co-payment for health services, and the high response consistency in those subjects who were re-interviewed, make the existence of the hypothetical bias less likely to happen, although it cannot be completely ruled out.

### Implications and conclusion

We find that these results have several implications for designing health policies. First, it is proven once again that the absence of payment at the moment of use does not make the user's perception of its value disappear. The recipient of public health services behaves as consumer choice theory predicts, valuing more, in economic terms, services taking more time, producing higher satisfaction, obtained under more pleasant circumstances, or implying a higher effort from the person providing it (such as home visit consultation). Income, level of education, age, and sex modify this perception of value, which has implications for populations that are predominantly female and in need of more care at an advanced age in life. On the other hand, there is a group of people who, although making use of the services and being as satisfied with them as the rest of users, feel they do not have the ability to pay at the moment of use, which could turn them into an excluded population in a different setting of service provision.

In addition, the perception of value is explained to a certain degree by the characteristics of the group of individuals enjoying them, and this influence is more important for extreme values of income distribution.

Both individual and inter-center variability should be of special interest for health organizations, since they would have to be taken into account for planning clinic and/or community interventions to a similar degree as when assigning resources. Knowing what determines the valuation users make of health services can also guide health managers in care-quality incentivisation policies, as the perception of value diminishes in identified groups.

Moreover, these differences in the expression of perceived value and how they adjust to economic theory can be considered a test of the usefulness of the CV method for eliciting preferences.

In conclusion, health services provided by the nurse in the framework of a public health system are perceived by the user as an element with real value in economic terms. The recipient of public health services behaves as a “rational” consumer, expressing different perceived values depending on the individual and environmental socioeconomic characteristics, on other personal characteristics, and on intrinsic attributes of the valued service.
